# Investigation of Thiocarbamates as Potential Inhibitors of the SARS-CoV-2 Mpro

**DOI:** 10.3390/ph14111153

**Published:** 2021-11-12

**Authors:** Katarzyna Papaj, Patrycja Spychalska, Katarzyna Hopko, Patryk Kapica, Andre Fisher, Markus A. Lill, Weronika Bagrowska, Jakub Nowak, Katarzyna Szleper, Martin Smieško, Anna Kasprzycka, Artur Góra

**Affiliations:** 1 Tunneling Group, Biotechnology Centre, Silesian University of Technology, Krzywoustego 8, 44-100 Gliwice, Poland; katarzyna.papaj@polsl.pl (K.P.); kapica.patrick@gmail.com (P.K.); weronika.bagrowska01@gmail.com (W.B.); kataszl203@student.polsl.pl (K.S.); 2 Biotechnology Centre, Silesian University of Technology, Krzywoustego 8, 44-100 Gliwice, Poland; walilko.patrycja@gmail.com (P.S.); katarzyna.hopko@polsl.pl (K.H.); anna.kasprzycka@polsl.pl (A.K.); 3 Computational Pharmacy, Department of Pharmaceutical Sciences, University of Basel, Klingelbergstrasse 61, 4056 Basel, Switzerland; and.fischer@unibas.ch (A.F.); markus.lill@unibas.ch (M.A.L.); martin.smiesko@unibas.ch (M.S.); 4 Department of Physical Biochemistry, Faculty of Biochemistry, Biophysics and Biotechnology, Jagiellonian University, Gronostajowa 7, 30-387 Krakow, Poland; kuba.nowak@uj.edu.pl; 5 Department of Chemistry, Silesian University of Technology, M. Strzody 9, 44-100 Gliwice, Poland

**Keywords:** Mpro, thiocarbamates, MST

## Abstract

In the present study we tested, using the microscale thermophoresis technique, a small library of thionocarbamates, thiolocarbamates, sulfide and disulfide as potential lead compounds for SARS-CoV-2 Mpro drug design. The successfully identified binder is a representative of the thionocarbamates group with a high potential for future modifications aiming for higher affinity and solubility. The experimental analysis was extended by computational studies that show insufficient accuracy of the simplest and widely applied approaches and underline the necessity of applying more advanced methods to properly evaluate the affinity of potential SARS-CoV-2 Mpro binders.

## 1. Introduction

The highly conserved region in the Mpro, the substrate-binding pocket, gives rise to a hope that compounds that inhibit the analogous protein in SARS-CoV-1 could also work on SARS-CoV-2 and provide a fast cure, for the treatment of the COVID-19 patients [1]. However, previous computational studies have shown that such a strategy can be insufficient [2,3]. Indeed, the initial attempts of known inhibitors repositioning have failed and none of the previously discovered compounds were applied in treatment [4,5,6,7].

In parallel, massive virtual screening campaigns were conducted, based on well-known databases of approved drugs, large drug-like molecules [8,9,10,11] as well as in-house small nature-based compounds [12,13,14,15], each providing a different set of potentially active compounds.

Besides pure computational studies, experimental screening techniques were applied. The fluorescence resonance energy transfer technique was used to test thiophene-2-carboxylate derivatives, quinoline derivatives, quercetins, peptidomimetics, anilides and others on the SARS-CoV2 Mpro [16]. Several groups have used a high-throughput X-ray crystallographic screening of drug libraries or small fragments against the SARS-CoV2-Mpro [17].

Despite over one year of battle against the COVID-19 pandemic, only a few examples of successful experimentally verified Mpro binders were reported [17,18,19,20]. Since the Mpro is a cysteine protease, the drug candidates are targeted toward cysteine residue. A good example is the first potential drug developed by Pfizer that has just started clinical trials [21]. In the structure of this drug, there are 4 amide bonds, including an intramolecular one. PF-07321332 compound features two five-membered rings (pyrrolidine and γ-lactam) as well as trifluoroamide, nitrile and alkyl moieties (tert-butyl and isopropyl) [22].

**Figure 1:**
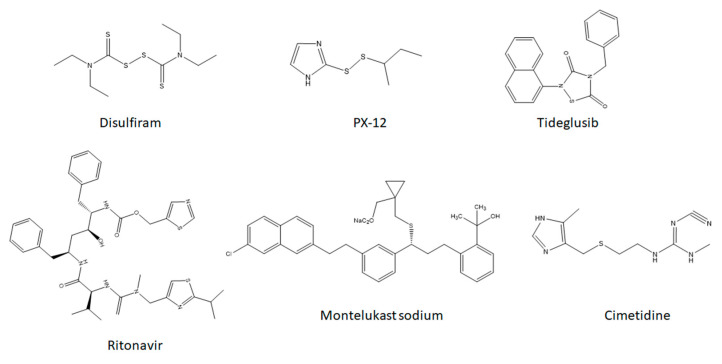
The structures of the compounds with known binding affinity.

The other one of the intensively tested groups of compounds targeting the active site of SARS-CoV2-Mpro are sulfur-containing derivatives, known also for their antiviral, antibacterial, cytotoxic or antiallergic activity [23,24]. This group includes compounds, such as: Disulfiram (IC50 = 9.35 ± 0.18 μM), PX-12 (IC50 = 21.39 ± 7.06 μM), Tideglusib (IC50 = 1.55 ± 0.30 μM), Ritonavir (IC50 = 13.7 ± 1.1 μM), Montelukast sodium (IC50 = 13.5 ± 1.0 μM), Cimetidine (Ki = ~3.27 μM) and others [9,17,25,26,27,28,29] (Figure 1).

**Figure 2:**
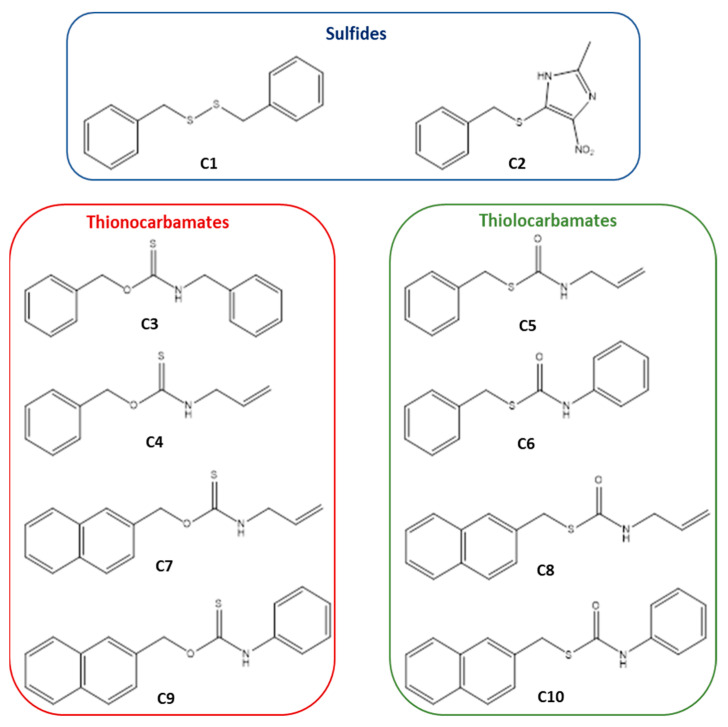
The structures of the tested compounds.

In this paper, we have applied the microscale thermophoresis technique to investigate the binding of a small library of in-house synthesized sulfur-containing compounds including thiocarbamates (thiono- and thiolocarbamates), sulfide and disulfide (Figure 2).

Interestingly, within the tested group, we have found one thionocarbamate with the binding affinity in the millimolar (mM) range, whereas corresponding thiolocarbamates did not show any binding to the SARS-CoV2-Mpro. To explain the reasons for such differences we have performed a series of in silico simulations, such as classical molecular docking, molecular dynamics and advanced MM/GBSA calculations. We found that docking procedures are unable to reproduce the binding affinity, but the MD-based MM/GBSA calculations show, that the thionocarbamate compound is capable to form an additional hydrogen bond and thus stabilize its position in the Mpro active site, whereas the corresponding thiolocarbamate tends to leave the active site. A rather simple structure of the best compound is advantageous as it can be used as a lead for optimization and in further search for potential SARS-CoV2-Mpro inhibitors.

## 2. Results

**Figure 3:**
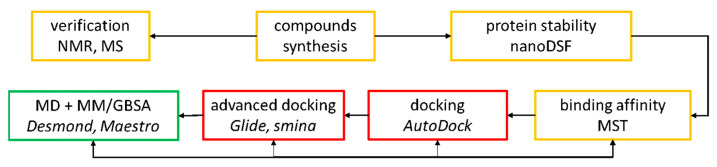
The workflow of experiments. Experimental studies (orange boxes) were followed by in silico analysis (unsuccessful-red boxes, successful green box) aiming for explanation of the observed results.

The general workflow of the experiments is summarized at Figure 3.

### 2.1. Thermal Stability Analysis

Prior to binding affinity experiments, the nanoDSF method was used to test the stability of the SARS-CoV-2 Mpro protein. The effect on the protein stability at different concentrations of DMSO (ranging from 0 to 5% *v*/*v*) and in the presence of the tested compounds was determined. The results indicate that the amount of DMSO in solution should be lower than 2.5% not to affect the protein stability. A higher concentration of DMSO (5% *v*/*v*) in solution lowers the Tm value from 55.8 °C to 54.83 °C (Supplementary Materials, Figure S1; Supplementary Materials, Table S1).

The effect of the compounds on the stability of the protein was examined with the highest possible concentration of the compounds in the solution limited by their solubility and DMSO threshold set at 1.25%. Among the compounds C1-C10, only 1 compound, namely C6 visibly affected protein stability in the highest possible concentration (250 μM), hence we had to decrease its concentration to 125 μM. Furthermore, we observed a slight effect of the C3 and C4 on the stability of the protein (Supplementary Materials, Figure S2; Supplementary Materials, Table S2).

### 2.2. The Binding Affinity of the Compounds to the SARS-CoV-2 Mpro Protein

The binding affinity of the compounds to the SARS-CoV-2 Mpro protein was measured during the MST experiment using a mixture of a labeled and unlabeled protein. This technique is based on the fluorescence of the tag, which was used to label the protein and thus requires none or very low intrinsic fluorescence of the ligands when compared to the analyzed protein. The native fluorescence of the compounds C1-C10 was measured and compared to the fluorescence of the mixture of the SARS-CoV-2 Mpro proteins. None of the compounds exhibited substantial fluorescence and thus they were used in other experiments in the whole tested concentration range.

**Figure 4:**
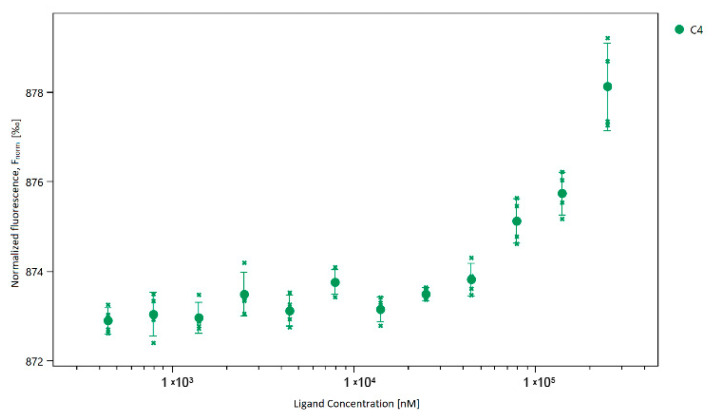
The binding curve of the compound C4 to the SARS-CoV-2 M^pro^.

The MST assay showed that only one compound (C4) binds to the SARS-CoV-2 Mpro protein (Figure 4). It is visible that the binding curves lack the points in the higher concentration range, which was not achievable due to the solubility limitations. For this reason, only the approximated value of the Kd 4 mM ± 15 mM was estimated.

### 2.3. Molecular Docking of Sulfur-Containing Compounds

All synthesized compounds were considered in the docking analysis. Initial docking was done with the AutoDock tools for four models: crystallographic structures without water molecules (6Y2E_no_wat, 6LU7_no_wat) and structures containing crystal water (6Y2E_wat, 6LU7_wat) (Supplementary Materials, Table S3). The docking box was defined by the active site amino acids: His41 and Cys145. The presence of the water molecules had no significant effect on binding affinity value. The presence of the N3 inhibitor in the crystal structure of the protein influenced the obtained results. Binding affinity was mostly lower for the 6LU7 protein structure than for 6Y2E. Most of the compounds were scored with a binding free energy of approximately −6.0 kcal/mol. Compounds with the best scores were: −8.7 kcal/mol for C6 ligand and 6LU7_no_wat receptor and −6.7 kcal/mol for C10 ligand and 6Y2E_no_wat receptor. Free energies obtained for the compounds with experimentally determined Kd (C4) were −6.1 kcal/mol for 6LU7 and −5.1 kcal/mol for 6Y2E, which does not correspond to the best score.

**Figure 5:**
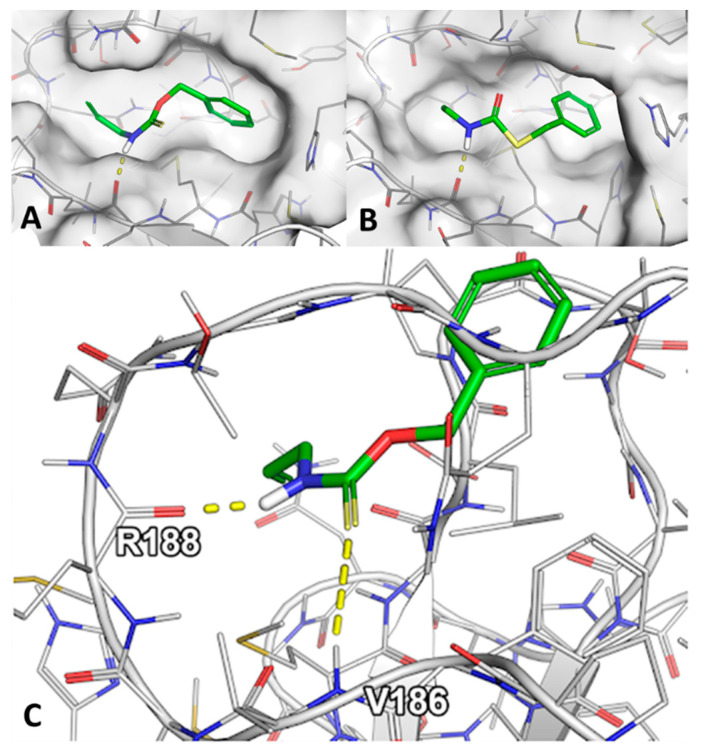
The comparison of the binding of the C4 and C5 compounds. (**A**) The best pose of the C4 compound (Glide docking). (**B**) The best pose of the C5 compound (Glide docking). (**C**) The last frame from the MD simulations of the Mpro protein with bound C4 compound showing an additional hydrogen bond. In case of the C5 compound, the ligand has left the binding site spontaneously.

Since the AutoDock docking procedure could not reproduce the experimental result we aimed for a more challenging docking protocol with prior validation regarding the prediction of crystallographically determined binding modes. Using both smina and Glide SP protocols, we established an ensemble of eight structures that could correctly reproduce 70.4% and 96.3% of 27 cocrystallized ligands within an RMSD (Root Mean Square Deviation) threshold of 2.5 Å, respectively. The smina docking scores ranged from −5.9 kcal/mol for C1 to −7.5 kcal/mol for C10, while the Glide scores ranged from −5.5 kcal/mol for C4 and C5 to −7.3 kcal/mol for C9 and C10. The docking scores from Glide and smina presented a Pearson correlation coefficient of 0.84 (Supplementary Materials, Table S4). After docking, the obtained complexes with Glide were subjected to classical MD simulations with a duration of 50 ns (explicit water, Desmond software) to observe whether the binding of the predicted inhibitors is subjective to spontaneous changes; additionally, the binding free energy of each ligand was quantified using the MM/GBSA protocol. The calculated energy values varied to a great extent and ranged from −29.1 to −49.0 kcal/mol for C5 and C8, respectively. Visual inspection of the trajectories with the closely related compounds C4 and C5 revealed the dissociation of the latter, while C4 presented an additional hydrogen bond in comparison to the initial starting conformation obtained from docking (Figure 5C).

## 3. Discussion

Sulfur-containing derivatives are well known for their antiviral and antibacterial activities and are often used against enzymes relying on catalytically active cysteine in their active site, such as the SARS-CoV2-Mpro. Therefore, we tested a small group of rather simple sulfur-containing compounds that could be used as a core structure for the design of novel inhibitors. We sought some initial binding affinity in the limited library of thiocarbamates (thiono- and thiolocarbamates), sulfide and disulfide using the microscale thermophoresis technique. The library was designed in such a way that all compounds can be further used as a lead and can be easily synthetically modified to provide a vast library of analogs. Due to a high similarity (homology) of the compounds, we aimed at elucidating fine differences in structural and electronic properties driving their affinity towards the active site of the SARS-CoV2-Mpro.

The measurement of the binding affinity yielded only a single hit, a representative of the thionocarbamate group with Kd estimated in the mM range. Interestingly, for a corresponding thiolocarbamate, no binding could be observed. Such situations allow the challenging of several in silico approaches to prove their applicability in the proper assignment of the compound affinity.

The computational methods are believed to significantly improve the success rate in discovering the initial binders.

However, one of the first in silico studies focused on the SARS-CoV2-Mpro 2 pointed out that this protein can be a challenging system for computational chemists and more advanced computational (and thus also time and cost-demanding) techniques are required to provide accurate results. Indeed, when we applied the conceptually simplest procedures for our small set, they failed in recognizing the active compound and the predicted binding affinity for the experimentally verified binder was among the worst ones (Supplementary Materials, Table S5). Therefore, we applied an advanced docking protocol followed by complex MM/GBSA calculations aiming to detect a correlation between the predicted binding free energies of the analyzed compounds and the observation that C4 is the only ligand among them with an affinity toward the dimeric form of Mpro. The advanced ensemble docking procedure provides much more reliable results than the classical docking protocol used in the first step [30], however, in our case both advanced protocols were unable to reproduce experimentally observed data. The more elaborate MD-based MM/GBSA calculations predicted C4 among the better binders. Interestingly, C4 presented the best ligand efficiency among all compounds assessed here. Even though ligand efficiency does not represent a measurable physical or thermodynamic property and depends on the reported concentration or energy unit, it can be a useful property in computational chemistry projects [31]. Due to the often strong influence of the van der Waals term in scoring functions and post-scoring methods, such as MM/GBSA [32], scaling the predicted affinities of comparable ligands with the number of heavy atoms can be advantageous [31]. Further, larger ligands with a high number of heavy atoms often suffer from a larger entropic penalty upon binding to a binding pocket due to the loss of more degrees of freedom associated with more rotatable bonds. Entropy is often treated insufficiently in the current scoring methodology, which is mostly focused on enthalpic contributions, even though it can largely contribute to the binding affinity and compensate (or even reverse) gains from enthalpically favorable interactions [33,34]. Thus, in the case of the congeneric ligands studied here, the improved ligand efficiency of C4 compared to the remaining compounds, stands in correlation with experiments suggesting this to be a valuable parameter in Mpro inhibitor design.

Due to the remarkable similarity between C4 and C5, which only differ by being an O-thiocarbamate and S-thiocarbamate respectively, their different binding behavior toward Mpro is especially interesting. While the binding modes obtained from molecular docking were inconclusive besides the cis-orientation of C4, the MD simulations started using the docking complexes presented significant differences among the ligands. During the 50 ns simulation, C5 dissociated from the enzyme while C4 formed an additional hydrogen bond to the protein (Figure 5). Thus, the time-evolved insight provided from these simulations offered a potential explanation for the experimentally observed affinity differences. Other similar ligands of C4 and C5, which did not present a measurable binding affinity, include their closely-related derivatives C3 and C6. In these ligands, the benzyl moiety was replaced with a phenyl group, which could not be accommodated at the same position without significantly perturbing the placement of the remaining ligand according to the obtained docking poses. This might explain the experimental behavior of these ligands.

A simple method of synthesizing thiocarbamates allows flexible substitution of both functional groups of the C4 compound and thus opens doors for substantial improvement of the solubility and binding properties of C4 derivatives. The aromatic ring can incorporate heteroatoms or can hold different electron-donating or withdrawing substituents which can improve binding without significant enlargement of the group fitting to the active site cavity. Similarly, the allyl moiety can be modified and provide donors or acceptors increasing the variability of the library of C4 compound derivatives.

In summary, our study highlights an interesting compound that can be used as a lead structure for the design of novel SARS-CoV2-MPro inhibitors. Its simple chemicals structure allows for facile future modification aiming for the improvement of binding affinity, solubility and pharmacokinetic properties. Additionally, our studies indicate the drawbacks in the application of the simplest in silico procedures for SARS-CoV2-MPro inhibitors discovery study.

## 4. Materials and Methods

### 4.1. Protein Preparation

The concentration of the purified SARS-CoV-2 Mpro protein was measured using the NanoDrop Spectrophotometer and BCA assay (Pierce™ BCA Protein Assay Kit, Thermo Fisher Scientific, Rockford, IL, USA) based on the beforehand prepared calibration curve on the bovine serum albumin. In the next step, a part of the Mpro protein was labeled using the Protein Labeling Kit RED-NHS 2nd Generation (NanoTemper Technologies, Munich, Germany) according to the manufacturer’s instructions. The concentration of the protein in the labeling mixture was adjusted to 10 μM and the molar dye:protein ratio was 3:1. The labeling reaction was performed in the labeling buffer NHS (130 mM NaHCO_3_, 50 mM NaCl, pH 8.2–8.3) at room temperature for 30 min in the dark. The unbound dye was removed using a dye removal column equilibrated with HEPES buffer. The degree of labeling (DOL parameter) was determined using UV/VIS spectrophotometry at 650 and 205 nm. The achieved DOL value was around 1.0. After the labeling, the labeled protein solution was supplemented with Pluronic F-127 with the final concentration of 0.01% (*w*/*v*).

### 4.2. Compounds Preparation

The compounds C1-C10 (synthesized in-house, characteristics of which are presented in the Supplementary Materials) [35,36,37] were dissolved in DMSO purged with Argon (due to their low solubility in water) to the final concentration of 20 mM.

### 4.3. Thermal Stability Analysis

#### 4.3.1. The Influence of DMSO Concentration on the SARS-CoV-2 Mpro Protein Stability

The 3 μM unlabeled SARS-CoV-2 Mpro solution was tested with a series of concentrations of DMSO (from 0 to 5% *v*/*v*) after 2 hr incubation at room temperature. The experiment was performed on Standard Capillaries Prometheus NT.48 (NanoTemper, Munich, Germany) in 2 technical repetitions using the Prometheus NT.48 apparatus with the following parameters: excitation power: 100%, initial temperature: 20 °C, final temperature: 80 °C and slope: 2 °C/min.

#### 4.3.2. Thermal Stability of the SARS-CoV-2 Mpro with the Examined Compounds

Each solution of the examined compounds or DMSO were mixed with 4 μM unlabeled SARS-CoV-2 Mpro solution in HEPES buffer with 0.01% (*w*/*v*) of Pluronic F-127 in separate samples. The final concentration of compound C8: 62.5 μM; C1, C6, C9, C10: 125 μM and C2, C3, C4, C5, C6, C7: 250 μM. The blank sample consisted of the Mpro protein with 1.25% DMSO and without any of the compounds.

The experiment was performed on Standard Capillaries Prometheus NT.48 in two technical repetitions using the Prometheus NT.48 apparatus with the following parameters: excitation power: 100%, initial temperature: 20 °C, final temperature: 70 °C and slope: 2 °C/min.

### 4.4. The Binding Affinity of the Compounds to the SARS-CoV-2 Mpro Protein

#### 4.4.1. The Intrinsic Fluorescence of the Compounds

The assessment of the fluorescence of the compounds was carried out based on the comparison of the fluorescence of the compound solutions in HEPES buffer with 0.01% (*w*/*v*) of Pluronic F-127 and the SARS-CoV-2 Mpro protein.

The final concentration of compound C8: 62.5 μM; C1, C6, C9 and C10: 125 μM, C2, C3, C4, C5, C7: 250 μM and the mixture of labeled and unlabeled SARS-CoV-2 Mpro protein solution: 0.075 μM and 4 μM, respectively. The experiment was performed on Standard Capillary Chips in 2 technical repetitions using the Monolith NT. Automated with the following parameters: excitation power: 40% Nano-RED, MST Power: medium, Before MST: 3 s, MST-On Time 10 s and After MST: 1 s.

#### 4.4.2. Binding Affinity Measurement-MST Experiment

During the MST experiments, the concentration of the proteins in solution was kept constant while the compounds were titrated. The dilution series of the compounds were prepared by applying a 3:1 ratio with initial concentrations of the compounds: C8: 125 μM; C1, C6, C9 and C10: 250 μM, C2, C3, C4, C5, C7: 500 μM. HEPES buffer supplemented with 0.01% (*w*/*v*) of Pluronic F-127 was used to dilute the stock solutions of the compounds to the initial concentrations (mentioned above). The same buffer with an appropriate amount of DMSO (2.5% *v*/*v*) was used as the dilution buffer in the dilution series. Next, a constant amount of the mixture of the proteins in the HEPES buffer without DMSO (concentration of the labeled protein: 0.150 μM, unlabeled protein: 8 μM) was added in 1:1 volume ratio to the respective diluted compounds resulting in the final concentration of the labeled protein: 0.075 μM, unlabeled protein: 4 μM and the final concentration of the compounds starting from C8: 62.5 μM; C1, C6, C9 and C10: 125 μM, C2, C3, C4, C5, C7: 250 μM.

The experiments were performed in 3 independent repetitions with the following parameters of the analysis: Standard Capillary Chips, excitation power: 40%, Nano-Red, MST Power: medium, Before MST: 3 s, MST-On Time 10 s and After MST: 1 s.

### 4.5. Molecular Docking of Sulfur-Containing Compounds

Two crystal structures of SARS-CoV-2 main protease: apo enzyme (PDB ID: 6Y2E) and enzyme bound to N3 inhibitor (PDB ID: 6LU7) were downloaded from the Protein Data Bank [38]. N3 ligand from the 6LU7 crystal structure was removed. Initial docking was performed using AutoDock tools [39] on four models: crystallographic structures without water molecules (6Y2E_no_wat, 6LU7_no_wat) and structures containing crystal water (6Y2E_wat, 6LU7_wat). H++ server [40] was used to protonate the protein structures using standard parameters and pH 7.4. The 3D structures of 10 ligands from the library of sulfur-containing compounds were prepared using the ChemAxon MarvinSketch 19.22.0 and OpenBabel 2.3.2 software [41]. The docking box was defined by the active site amino acids: His41 and Cys145. We retained the ten best-scored poses from docking.

Next, we validated the Glide standard-precision (SP) [42] and smina [43] protocols regarding their capability to reproduce crystallographic binding modes in an ensemble docking setting. We evaluated ensembles of up to eight structures from 39 crystal structures with 27 non-covalent cocrystallized ligands at an RMSD threshold of 2.5 Å. The best-performing ensemble was retained for further procedures. For this procedure, the protein structures were treated with the Protein Preparation Wizard [44] in the Schrodinger Small-Molecule Drug Discovery Suite [45] with default specifications except for a pH value of 7.4 for steps concerning protonation. For the following production phase, we preprocessed the 10 ligand structures with the LigPrep [46] routine in Maestro to obtain energy-minimized 3D conformers with the OPLS3e force field and protonation states at pH 7.4 were predicted with Epik. We then docked the ligands to the selected ensemble and retained complexes with the lowest binding free energy of each compound (Glide score). The Pearson correlation coefficient between the docking scores from Glide and smina was computed using the pearsonr routine included in the scipy python package. Subsequent molecular dynamics (MD) simulations were conducted with the Desmond (v2019-1) [47] engine using the OPLS_2005 force field in an NPT ensemble. While the temperature was controlled by the Nose–Hoover thermostat, atmospheric pressure was maintained by the Martyna–Tobias–Klein barostat. Long-range forces were treated with the u-series algorithm [48] with a cutoff of 9 Å for short-range interactions. Bonds to hydrogen atoms were constrained with the M-SHAKE algorithm. The orthorhombic periodic systems were solvated with TIP3P water molecules and an appropriate number of counter ions was added to equalize the net system charge to zero. After the default relaxation protocol, every complex was simulated for 50 ns at 310 K. By default, the time step of the RESPA integrator was set to 2 fs. Atomic coordinates were saved at an interval of 10 ps. The MD simulations were then processed with the Molecular Mechanics Generalized Born Surface Area (MM/GBSA) protocol using the therma_mmgbsa.py script included in Maestro to obtain binding free energies for each ligand. We selected every second frame of the last 10 ns to be processed by the routine and averaged the final energies (triplicas). To determine the “ligand efficiency” descriptor values of each compound, we divided the calculated binding energy values as well as the docking scores by the number of heavy atoms.

## Data Availability

Data are contained within the article or Supplementary Materials.

## Supplementary Materials

The following are available online at https://www.mdpi.com/article/10.3390/ph14111153/s1, Chemical characteristics and information about synthesis of C1-C10 compounds, Table S1: The measured melting temperatures of SARS-CoV-2 M^pro^ in HEPES buffer with different DMSO concentration, Table S2: The measured melting temperatures of SARS-CoV-2 M^pro^ in HEPES buffer with tested compounds, Table S3: Binding affinities [kcal/mol] of sulfur-containing compounds, Table S4: Results from ensemble docking, Table S5.: Results from molecular docking with smina and Glide, as well as binding free energy obtained from the MM/GBSA protocol and the related ligand efficiency. The ligand efficiency was determined by dividing ΔG_MM/GBSA_ by the number of heavy atoms of the respective ligand, Figure S1: The comparison of the stability of SARS-CoV-2 M^pro^ in buffer and with different DMSO concentration, Figure S2: The analysis of the influence of the compounds C1-C10 on the thermostability of the SARS-CoV-2 M^pro^.
