# Supplementary material for: Investigation of Thiocarbamates as Potential Inhibitors of the SARS-CoV-2 Mpro
Source: Pharmaceuticals (Basel). 2021 Nov 12;14(11):1153. doi: 10.3390/ph14111153 (PMC8621115; doi:10.3390/ph14111153)
Supplement: Supplementary file 1 [file pharmaceuticals-14-01153-s001.zip › pharmaceuticals-1415857-supplementary.pdf]

Supporting data for:

Investigation of thiocarbamates as potential inhibitors of the SARS-CoV-2 Mpro

Katarzyna Papaj [1], Patrycja Spychalska [2], Katarzyna Hopko [2], Patryk Kapica [1], Andre Fisher [3], Markus A. Lill [3], Weronika Bagrowska [1], Jakub Nowak [4], Katarzyna Szleper [1], Martin Smieško [3], Anna Kasprzycka [2,5], and Artur Góra [1,*]

[1] Tunneling Group, Biotechnology Centre, Silesian University of Technology, Krzywoustego 8, 44-100 Gliwice, Poland; katarzyna.papaj@polsl.pl (K.P.); kapica.patrick@gmail.com (P.K.); weronika.bagrowska01@gmail.com (W.B.); kataszl203@student.polsl.pl (K.Sz.); a.gora@tunnelinggroup.pl (A.G.)

[2] Biotechnology Centre, Silesian University of Technology, Krzywoustego 8, 44-100 Gliwice, Poland; walilko.patrycja@gmail.com (P.S.); katarzyna.hopko@polsl.pl (K.H.); anna.kasprzycka@polsl.pl (A.K.)

[3] Computational Pharmacy, Department of Pharmaceutical Sciences, University of Basel, Klingelbergstrasse 61, 4056 Basel, Switzerland; and.fischer@unibas.ch (A.F.); markus.lill@unibas.ch (M.A.L.); martin.smiesko@unibas.ch (M.S.)

[4] Department of Physical Biochemistry, Faculty of Biochemistry, Biophysics and Biotechnology, Jagiellonian University, Gronostajowa 7, 30-387 Krakow, Poland; kuba.nowak@uj.edu.pl (J.N.)

[5] Department of Chemistry, Silesian University of Technology, M. Strzody 9,44-100 Gliwice, Poland;

[*] e-mail: a.gora@tunnelinggroup.pl, artur.gora@polsl.pl phone: +48 32 237 16 59, Tunneling Group, Biotechnology Centre, Silesian University of Technology, Krzywoustego 8, 44-100 Gliwice, Poland

Experimental procedure for obtaining compounds C1-C10

General. All solvents were purified and dried according to standard methods prior to use. Reagents were purchased from commercial sources and were used without further purification. Analytical thin layer chromatography (TLC) was performed on precoated plates of silica gel 60 F_{254} (Merck) and visualized by exposure to ultraviolet light, or an acid solution of pallad chloride. Chromatographic purification was performed on silica gel 60 (Merck) 0.063-0.2 mm. The ^{1}H-NMR spectra were recorded on Varian 300 MHz or Varian 600 MHz and the ^{13}C-NMR spectra were recorded at 100 or 150 MHz. Chemical shifts are given relative to TMS or the appropriate solvent peak. Mass spectra were recorded at ESI Mass Spectrometer ABSciex System 4000 QTRAP® at positive mode of ionization.

1,2-Dibenzyldisulfane (**C1**)

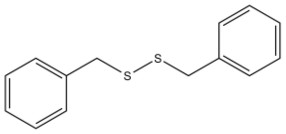

Obtained according to a literature procedure with yield 78%[35].

^{1}H-NMR (400MHz, $CDCl_3$): 7.34-7.22 (m, 10H, Ph), 3.60 (s, 4H, $-C\underline{H}_2-$). **^{13}C-NMR** (100MHz, $CDCl_3$): 137.5 (C-1), 129.6 (C-2, C-6), 128.6 (C-3, C-5), 127.6 (C-4), 43.4 ($-\underline{C}H_2-$); **ESI-MS**: *m/z* 269.26 $[M+Na]^+$ (*m/z calc.* 269.04 $[M+Na]^+$).

2-Methyl-4-nitro-5-[(phenylmethyl)thio]-1H-imidazole (**C2**)

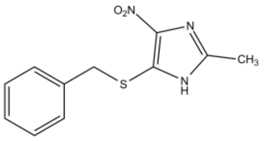

Obtained according to a literature procedure with yield 60%. [35].

^{1}H-NMR (600MHz, $CDCl_3$): 7.28-7.18 (m, 5H, Ph), 4.94 (bs, 1H, $-N\underline{H}-$), 4.30 (d, 2H, *J*=5.4Hz, $-CH_2-$), 3.63 (s, $-CH_3$); **^{13}C-NMR** (150MHz, $CDCl_3$): 156.6 ($-\underline{C}-NO_2$), 137.0 (Ph), 134.0 ($-NH-\underline{C}(S)=$), 133.1 (Ph) 128.9 (Ph), 128.8 (Ph), 127.6 (Ph), 45.3 ($-CH_2-$), 15.9 ($-CH_3$); **ESI-MS**: *m/z* 272.38 $[M+Na]^+$ (*m/z calc.* 272.28 $[M+Na]^+$).

Benzyl N-benzylthiocarbamate (**C3**)

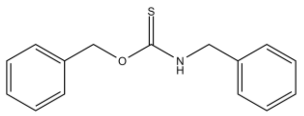

Obtained according to a literature procedure with yield 80% [36].

^{1}H-NMR (600MHz, $CDCl_3$): 7.20 (m, 10H, Ph), 6.54 (s br, 1H, $-N\underline{H}-$), 5.50 (s, 2H, $-O-C\underline{H}_2-Ph$), 4.76 (d, 1H, *J*=5.4Hz, $-NH-C\underline{H}_2-Ph$); **^{13}C-NMR** (150MHz, $CDCl_3$): 190.4 ($\underline{C}=S$), 136.8 ($-O-CH_2-\underline{C\text{-}1}$(Ph)), 135.8 ($-NH-CH_2-(\underline{C\text{-}1})$Ph), 127.8-129.0 (10C, Ph), 72.1 ($-O-\underline{C}H_2-Ph$), 49.4 ($-NH-\underline{C}H_2-Ph$); **ESI-MS**: *m/z* 280.31 $[M+Na]^+$ (*m/z calc.* 280.08 $[M+Na]^+$).

Benzyl N-allylthiocarbamate (**C4**)

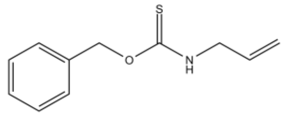

Obtained according to a literature procedure with yield 89% [36].

^{1}H-NMR (400MHz, $CDCl_3$): 7.40-7.33 (m, 5H, Ph), 6.36 (bs, 1H, -N$\underline{H}$-), 5.84 (m, 1H, -CH_2-C$\underline{H}$=CH_2), 5.48 (s, 2H, -C$\underline{H_2}$-Ph), 5.15-5.27 (m, 2H, -CH_2-CH=C$\underline{H_2}$), 4.22 (tt, 2H, *J*=1.6Hz, 6.4Hz, -C$\underline{H_2}$-CH=CH_2); **^{13}C-NMR** (100MHz, $CDCl_3$): 190.4 ($\underline{C}$=S), 135.9 ($\underline{C\text{-}1}$, Ph), 132.7 (-CH_2-$\underline{C}$H=CH_2), 127.3-128.9 (5C, Ph), 117.7 (-CH_2-CH=$\underline{C}H_2$), 72.2 (-$\underline{C}H_2$-CH=CH_2), 47.9 (-$\underline{C}H_2$-Ph)); **ESI-MS**: *m/z* 230.25 $[M+Na]^+$ (*m/z calc.* 230.06 $[M+Na]^+$).

S-benzyl N-benzylcarbamothioate (**C5**)

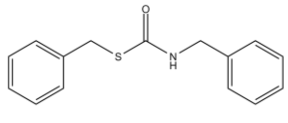

Obtained according to a literature procedure with yield 97% [37].

^{1}H-NMR (600MHz, $CDCl_3$): 7.34-7.24 (m, 10H, Ph), 5.60 (bs, 1H, -N$\underline{H}$-), 4.47 (s, 2H, -NH-C$\underline{H_2}$-Ph), 4.18 (s, 2H, -S-C$\underline{H_2}$-Ph); **^{13}C-NMR** (150MHz, $CDCl_3$): 167.0 ($\underline{C}$=S), 138.4 (-NH-CH_2-$\underline{C\text{-}1}$(Ph)), 137.7 (-S-CH_2-$\underline{C\text{-}1}$(Ph)), 127.3-129.0 (10C, Ph), 45.6 (-NH-$\underline{C}H_2$-Ph), 34.4 (-S-$\underline{C}H_2$-Ph); **ESI-MS**: *m/z* 280.11 $[M+Na]^+$ (*m/z calc.* 280.08 $[M+Na]^+$).

S-benzyl N-allylcarbamothioate (**C6**)

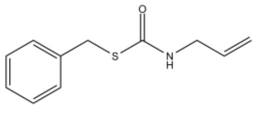

Obtained according to a literature procedure with yield 90% [37].

^{1}H-NMR (600MHz, $CDCl_3$): 7.23-7.34 (m, 5H, Ph), 5.79-5.86 (m, 1H, -CH_2-C$\underline{H}$=CH_2), 5.40 (bs, 1H, -N$\underline{H}$-), 5.17-5.22 (m, 2H, -CH_2-CH=C$\underline{H_2}$), 4.17 (s, 2H, -C$\underline{H_2}$-Ph), 3,92 (bs, 2H, -C$\underline{H_2}$-CH=CH_2); **^{13}C-NMR** (150MHz, $CDCl_3$): 166.9 ($\underline{C}$=O), 138.4 ($\underline{C\text{-}1}$(Ph)), 133.8 (-CH_2-$\underline{C}$H=CH_2), 129.0-127.3 (5C, Ph), 117.1 (-CH_2-CH=$\underline{C}H_2$), 43.9 (-$\underline{C}H_2$-CH=CH_2), 34.4 (-$\underline{C}H_2$-Ph); **ESI-MS**: *m/z* 230.19 $[M+Na]^+$ (*m/z calc.* 230.06 $[M+Na]^+$).

Naphtyl N-allylthiocarbamate (**C7**)

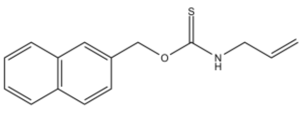

Obtained according to a literature procedure with yield 82% [36].

^{1}H-NMR (600MHz, $CDCl_3$): 7.82-7.85 (m, 4H, Naph), 7.47-7.50 (m, 3H, Naph), 6.40 (bs, 1H, -N$\underline{H}$-), 5.85-5.92 (m, 1H, -CH_2-C$\underline{H}$=CH_2), 5.64 (s, 2H, -C$\underline{H_2}$-Naph), 5.15-5.26 (m, 2H, -C$\underline{H_2}$-CH=CH_2), 4.22 (t, 2H, *J*=6Hz, -CH_2-CH=C$\underline{H_2}$); **^{13}C-NMR** (150MHz, $CDCl_3$): 190.4 ($\underline{C}$=S), 133.2 (-CH_2-$\underline{C}$H=CH_2), 132.9-125.9 (10C, Naph), 117.7 (-$\underline{C}H_2$-CH=CH_2), 72.3 (-$\underline{C}H_2$-Naph), 47.9 (-$\underline{C}H_2$-CH=CH_2); **ESI-MS**: *m/z* 280.22 $[M+Na]^+$ (*m/z calc.* 280.08 $[M+Na]^+$).

S-naphtyl N-allylcarbamothioate (**C8**)

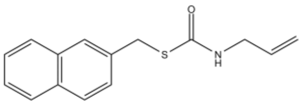

Obtained according to a literature procedure with yield 95% [37].

^{1}H-NMR (600MHz, CDCl$_3$): 7.78-7.81 (m, 4H, Naph), 7.44-7.46 (m, 3H, Naph), 5.78-5.88 (m, 1H, -CH$_2$-CH=CH$_2$), 5.37 (bs, 1H, -NH-), 5.22-5.16 (m, 2H, *J*=10.2Hz, 16.8Hz, -CH$_2$-CH=CH$_2$), 4.34 (s, 2H, -CH$_2$-Naph), 3.93 (bs, 2H, -CH$_2$-CH=CH$_2$); **^{13}C-NMR** (150MHz, CDCl$_3$): 163.9 (C=O), 133.5 (-CH$_2$-CH=CH$_2$), 126.0-132.7 (10C, Naph), 117.1 (-CH$_2$-CH=CH$_2$), 44.0 (-CH$_2$-CH=CH$_2$), 34.7 (-CH$_2$-Naph); **ESI-MS**: *m/z* 280.22 [M+Na]$^+$ (*m/z calc.*: 280.08 [M+Na]$^+$).

Naphtyl N-benzylthiocarbamate (**C9**)

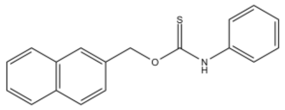

Obtained according to a literature procedure with yield 85% [36].

^{1}H-NMR (600MHz, CDCl$_3$): 7.14–7.85 (m, 12H, Ar), 5.80 (bs, 1H, -NH-), 5.66 (s, 2H, -CH$_2$-Naph); **^{13}C-NMR** (150MHz, CDCl$_3$): 188.6 (C=S), 133.9-126.6 (16C, Ar), 73.2 (-CH$_2$-Naph); **ESI-MS**: *m/z* 316.31 [M+Na]$^+$ (*m/z calc*. 316.08 [M+Na]$^+$).

S-naphtyl N-phenylcarbamothioate (**C10**)

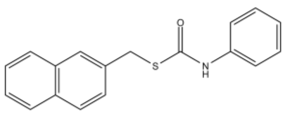

Obtained according to a literature procedure with yield 90% [37].
^{1}H-NMR (600MHz, CDCl$_3$): 7.06-7.80 (m, 12H, Ar), 3.52 (bs, 1H, -NH-); **^{13}C-NMR** (150MHz, CDCl$_3$): 165.3 (C=O), 135.3-125.9 (16C, Ar), 34.8 (-CH$_2$-Naph); **ESI-MS**: *m/z* 316.40 [M+Na]$^+$ (*m/z calc.*: 316.08 [M+Na]$^+$**Table S1.** The measured melting temperatures of SARS-CoV-2 Mpro in HEPES buffer with different DMSO concentration.

Analysed system	Additives	Tm [°C]	ΔTm [°C]
	0% DMSO	55.80°C	±0.02°C
SARS-CoV-2	0.5% DMSO	55.69°C	±0.05°C
Mpro	1% DMSO	55.53°C	±0.08°C
	2.5% DMSO	55.16°C	±0.04°C
	5% DMSO	54.83°C	±0.01°C

Table S2. The measured melting temperatures of SARS-CoV-2 Mpro in HEPES buffer with tested compounds.

Analysed system	Buffer	Additives	Tm [°C]	ΔTm [°C]
		1.25% DMSO	55.39°C	±0.07°C
		125 µM of C1	55.38°C	±0.03°C
		250 µM of C2	55.30°C	±0.04°C

SARS-CoV-2 M pro	HEPES	250 μM of C3	54.87°C	±0.06°C
		250 μM of C4	54.85°C	±0.06°C
		250 μM of C5	55.22°C	±0.05°C
		250 μM of C6	54.60°C	±0.06°C
		250 μM of C7	55.09°C	±0.03°C
		62.5 μM of C8	55.53°C	±0.12°C
		125 μM of C 9	55.53°C	±0.02°C
		125 μM of C10	55.59°C	±0.03°C

Table S3. AutoDock binding affinities [kcal/mol] of sulfur-containing compounds

Compound	6LU7_no_wat	6LU7_wat	6Y2E_no_wat	6Y2E_wat
C1	-4,4	-5,5	-4,4	-4,4
C2	-7,1	-7,0	-6,1	-6,1
C3	-6,3	-6,1	-4,7	-4,7
C4	-6,1	-6,1	-5,1	-5,1
C5	-6,7	-6,6	-4,9	-4,7
C6	-8,7	-8,2	-5,7	-5,7
C7	-8,3	-8,2	-5,6	-5,7
C8	-7,1	-7,1	-5,3	-5,3
C9	-5,1	-5,1	-5,4	-5,4
C10	-5,3	-5,3	-6,7	-6,6

Table S4. Results from ensemble docking.

Ensemble size	Percentage	Protocol
4	81.5%	Glide SP
	63.0%	smina
5	85.2%	Glide SP
	66.7%	smina
6	88.9%	Glide SP
	70.4%	smina
7	92.6%	Glide SP
	70.4%	smina
8	96.3%	Glide SP

Table S5. Results from molecular docking with smina and Glide, as well as binding free energy obtained from the MM/GBSA protocol and the related ligand efficiency. The ligand efficiency was determined by dividing ΔGMM/GBSA by the number of heavy atoms of the respective ligand.

Compound	smina score (kcal/mol)	Glide score (kcal/mol)	ΔGMM/GBSA (kcal/mol)	Ligand efficiencya
C1	-5.9	-6.7	-29.4	-1.84
C2	-6.5	-5.9	-47.9	-2.82
C3	-6.6	-7.2	-47.7	-2.65
C4	-5.4	-5.5	-44.3	-3.16
C5	-5.4	-5.5	-29.1	-2.08
C6	-6.6	-6.5	-44.2	-2.60
C7	-6.3	-6.3	-35.3	-1.96
C8	-6.3	-6.1	-49.0	-2.72
C9	-7.2	-7.3	-30.0	-1.43
C10	-7.5	-7.3	-35.5	-1.69

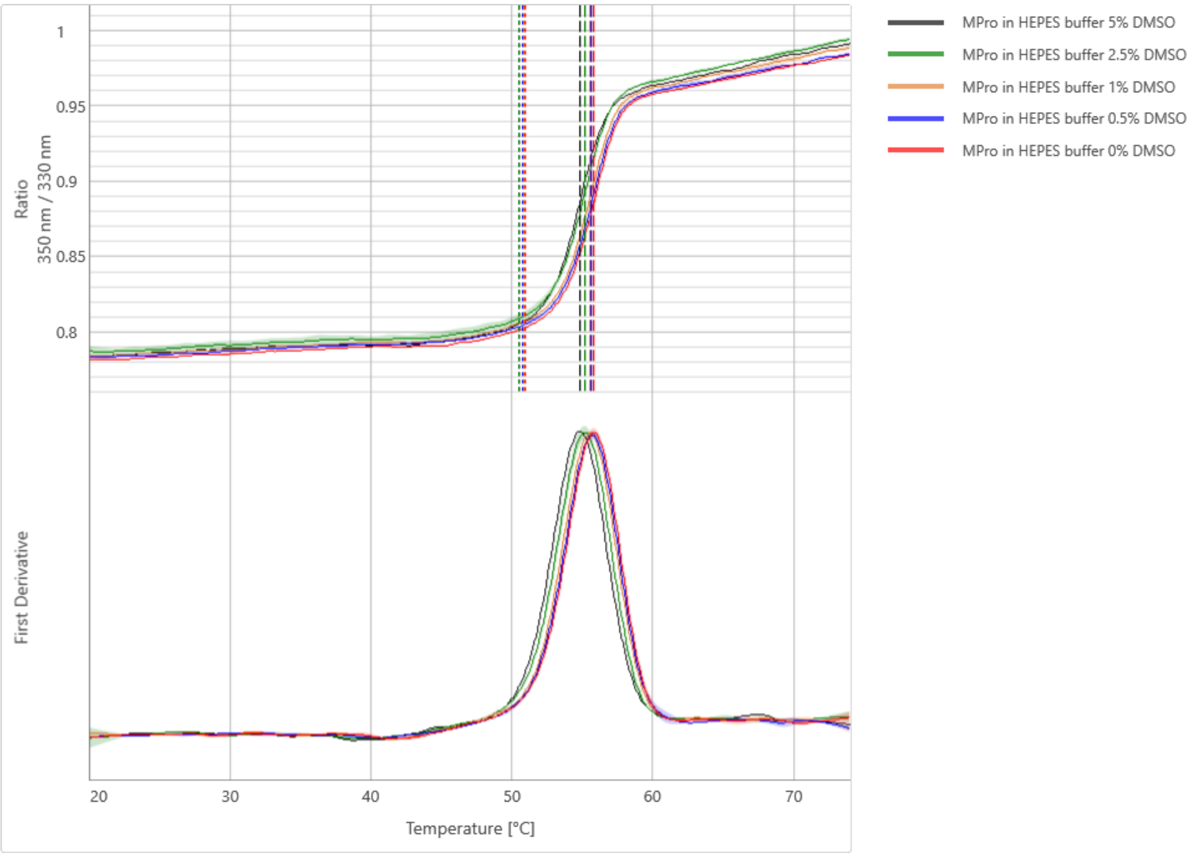

Figure S1. The comparison of the stability of SARS-CoV-2 Mpro in buffer and with different DMSO concentration.

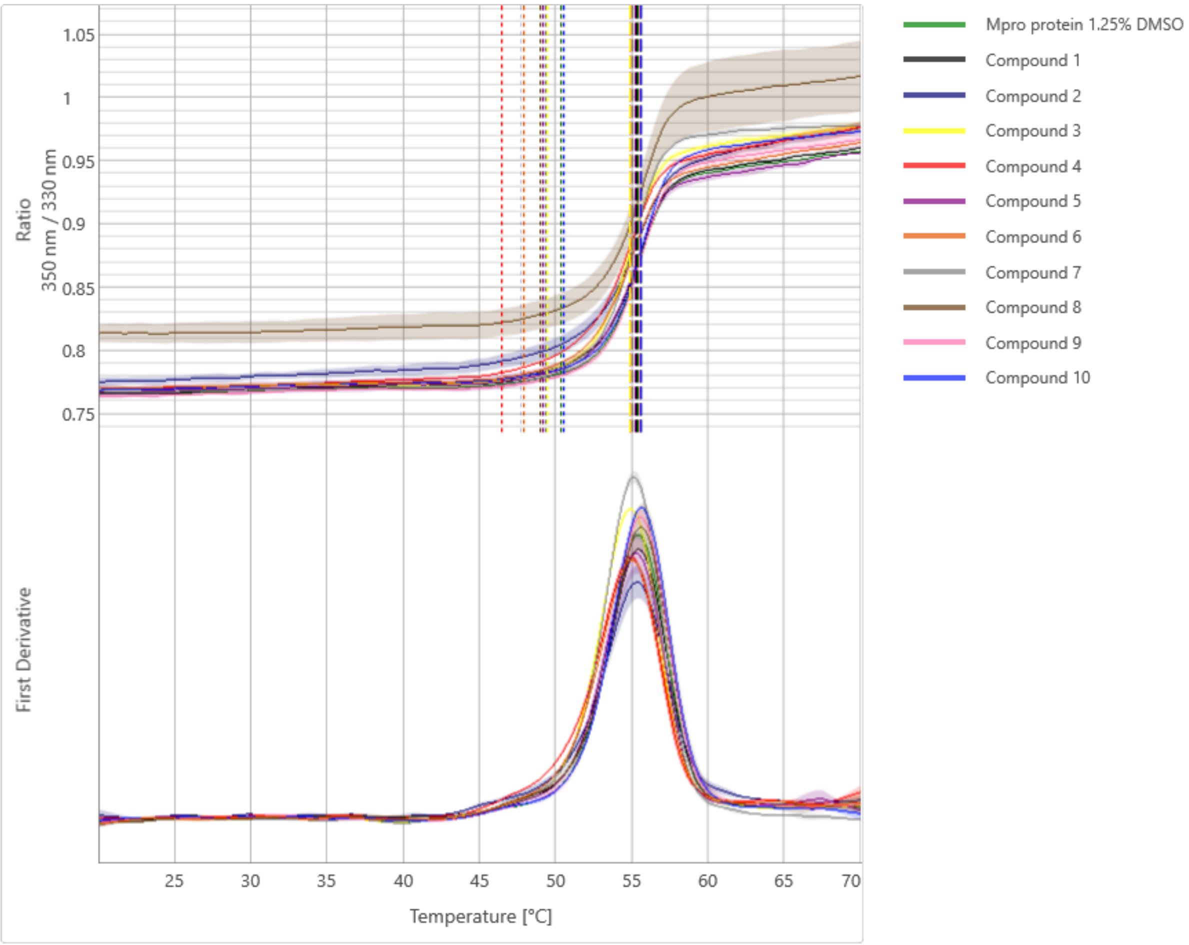

Figure S2. The analysis of the influence of the compounds C1-C10 on the thermostability of the SARS-CoV-2 Mpro.

References

35. Choudhuri, K.; Pramanik, M.; Mal, P. Direct C–S Bond Functionalization of Benzyl Mercaptan. *Eur J Org Chem.* **2020**, *25*, 3906–3913. https://doi.org/10.1002/ejoc.202000521
36. Kasprzycka, A.. Ptaszek-Budniok, A.. Szeja, W. Simple and efficient method for the protection of hydroxyl groups as 4-methoxybenzyl ethers. *Synth. Commun.*, **2014**, 44, 2276-2284. https://doi.org/10.1080/00397911.2014.894526
37. Komor, R.; Kasprzycka, A.; Pastuch-Gawołek, G.; Szeja, W. Simple synthesis of glycosylthiols and thioglycosides by rearrangement of O-glycosyl thionocarbamates. *Carbohydr Res.* **2014**, *396*, 37–42. https://doi.org/10.1016/j.carres.2014.07.001
